# Successful Treatment of Tumor-Induced Osteomalacia by Multidisciplinary Therapy with Radiation to Intracranial Fibromyxoid Tumor

**DOI:** 10.1155/2021/8841259

**Published:** 2021-06-14

**Authors:** Mariangela Massaccesi, Francesco Miccichè, Mario Rigante, Gianluigi Petrone, Elisabetta Lepre, Maria Antonietta Gambacorta, Vincenzo Valentini

**Affiliations:** ^1^Fondazione Policlinico Universitario “A. Gemelli” IRCCS, Unità Operativa Complessa di Radioterapia, Dipartimento di Diagnostica per Immagini, Radioterapia Oncologica ed Ematologia, Roma, Italy; ^2^Fondazione Policlinico Universitario “A. Gemelli” IRCCS, Unità Operativa Complessa di Otorinolaringoiatria, Dipartimento di scienze dell'invecchiamento, Neurologiche, Ortopediche e della testa-collo, Roma, Italy; ^3^Fondazione Policlinico Universitario “A. Gemelli” IRCCS, Unità Operativa Complessa di Anatomia Patologica, Dipartimento di scienze della salute della donna, del bambino e di sanità pubblica, Roma, Italy; ^4^Università Cattolica del Sacro Cuore Facoltà di Medicina e Chirurgia, Roma, Italy

## Abstract

**Background:**

Tumor-induced osteomalacia (TIO) is a rare paraneoplastic syndrome caused by protein fibroblast-growth-factor-23 (FGF-23) secreting tumors. Complete tumor resection is the current standard of care for TIO; however, some patients may develop tumor recurrence. Due to the rarity of this paraneoplastic syndrome, the role of radiotherapy is unclear. This case is worth reporting because it adds to our knowledge some insights about the potential role of radiotherapy in this rare condition. *Case Presentation*. After multidisciplinary consultation, in July 2015, postoperative adjuvant radiotherapy was offered to a 52-year-old man with a multiple recurrent ossifying fibromyxoid tumor in the right frontal sinus causing TIO. The patient had a history of multiple bone fractures and pain since more than 20 years. The tumor had been removed in 2003 for the first time. Subsequent endoscopic resections of the tumor had been performed for recurrences of TIO in May 2012, October 2013, and July 2015. Starting from October 2015, external beam radiotherapy was delivered with a volumetric modulated arc technique to the tumor bed with a daily dose of 2 Gy up to a total dose of 60 Gy. After five years from treatment, the patient is free from local tumor relapse, TIO progression, and radiation-induced side effects.

**Conclusions:**

Radiotherapy may provide long-term TIO remission and tumor control, thus being a treatment option in cases where surgery is unfeasible or unsuccessful.

## 1. Introduction

Tumor-induced osteomalacia (TIO) or oncogenic osteomalacia is a rare paraneoplastic syndrome resulting from excessive production of the protein fibroblast growth factor-23 (FGF-23). FGF-23 act as a phosphaturic hormone and also favors inactivation of 1, 25 dihydroxyvitamin D, thus leading to compensatory hyperparathyroidism. Patients typically present with symptoms of gradually progressive weakness, bone pain, and pathological fractures. Benign mesenchymal or mixed connective tissue tumor is the most common histological feature of neoplasms associated with TIO. Complete tumor resection is the current standard of care for TIO [1]. However, some patients may develop tumor recurrence and benefit from a second excision [2]. Radiotherapy has been rarely used in case of inoperable tumors or incomplete resections [3–9]. We report the case of a patient who had a multiple recurrent ossifying fibromyxoid tumor in the left frontal sinus causing TIO.

## 2. Case Presentation

The patient was a 52-year-old man with no family history of cancer and no significant comorbidity except hypercholesterolemia and arterial hypertension.

The patient had a history of bone pain, weakness, and multiple pathological fractures for more than 20 years. In 2003, osteomalacia was diagnosed by bone biopsy at the level of the right knee. In the same period, the patient underwent also surgical removal of a small lesion on the left frontal sinus that was diagnosed as an ossifying fibromyxoid tumor. After surgery, the patient experienced a period of symptom improvement, but again, over the following years, bone pain progressively worsened, and many nontraumatic bone fractures occurred in the right femur, ribs, right tibia, right fibula, and metatarsal bones despite therapy with vitamin D3 and calcium. In 2012, the diagnostic hypothesis of osteomalacia of oncogenic origin finally arose, and the patient underwent targeted investigations.

A total body computed tomography (CT) scan documented the presence of dense tissue in the left frontal sinus. At that level, a magnetic resonance (MR) examination showed a lobulated lesion measuring 2 cm, which was hypointense in the T1 sequences and hyperintense in the T2 sequences and had intense contrast enhancement. The lesion highly expressed somatostatin receptors on a ^68^Ga-DOTATATE positron emission tomography (PET) scan. Blood tests documented low levels of phosphate and high levels of FGF-23.

**Figure 1:**
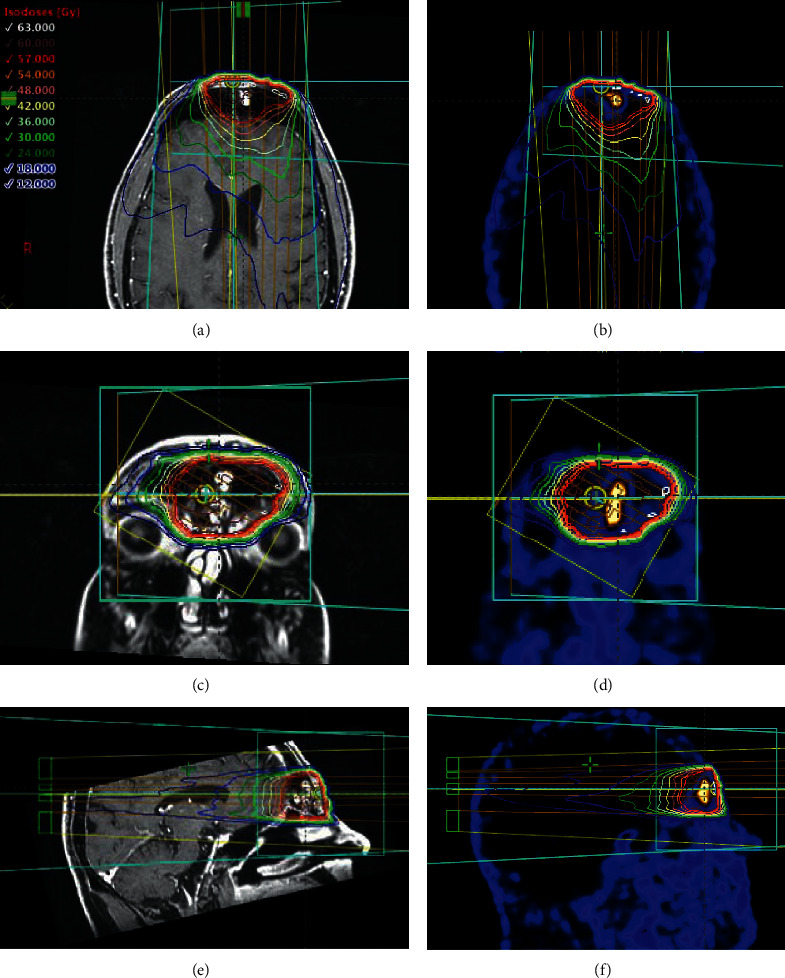
Dose distribution of postoperative VMAT for a multiple recurrent ossifying fibromyxoid tumor of the left frontal sinus causing TIO. Isodose lines are shown on axial (a, b), coronal (c, d), and sagittal (e, f) planes of coregistered preoperative MR (a, c, and e), ^68^Ga-DOTATATE PET, and simulation CT images (b, d, and f). The red line represents the 95% isodose.

In May 2012, the lesion of the left frontal sinus was removed through an endoscopic approach. Histological examination documented an ossifying fibromyxoid tumor that was framed as a recurrence of the lesion that had been removed in 2003. After a brief period of clinical and laboratory remission, TIO symptoms reappeared and gradually worsened. In April 2013, a ^68^Ga-DOTATATE PET scan showed a small lesion in the left frontal sinus with hyperexpression of somatostatin receptors. Six months later, a MR scan confirmed the presence of a contrast-enhancing lesion measuring 1.5 cm in the medial wall of the left frontal sinus, without signs of bone or orbital infiltration. In October 2013, the patient underwent an endoscopic frontal senectomy to remove the recurrence of the ossifying fibromyxoid tumor. Again, after less than two years of clinical remission, TIO symptoms worsened. In March 2015, an MR scan confirmed the presence of a small recurrence of the tumor, without signs of bone or orbital infiltration. A fourth surgical intervention was performed in July 2015 through a combined endoscopic and open approach to completely remove the lesion. A postoperative contrast-enhanced computed tomography was performed four weeks later, and it did not show any suspect sign of disease persistence. In August 2015, the case was discussed in a multidisciplinary tumor board and adjuvant radiation therapy was proposed to reduce the risk of further tumor recurrence. This multidisciplinary decision was shared with the patient who gave his consent to the treatment. Starting from October 2015, external beam radiotherapy was delivered with a volumetric modulated arc technique (VMAT) to the tumor bed with a daily dose of 2 Gy up to a total dose of 60 Gy. The patient was treated in the supine position with a thermoplastic facemask. The tumor bed was identified on coregistered preoperative MR, ^68^Ga-DOTATATE PET, and planning CT simulation images. The clinical target volume (CTV) included the tumor bed plus 1 cm margin along the frontal sinus walls. The planning target volume (PTV) included the CTV plus 3 mm margin. The organs at risk (OARs) were the optic chiasm, right and left cochlea, right and left lens, right and left eyeballs, and right and left optic nerves. Details of the radiation treatment are shown in Figure 1 and Table 1. Orthogonal planar kilo-voltage images were used daily before treatment as image guidance. Radiotherapy was completed without any treatment-related side effects.

**Figure 2:**
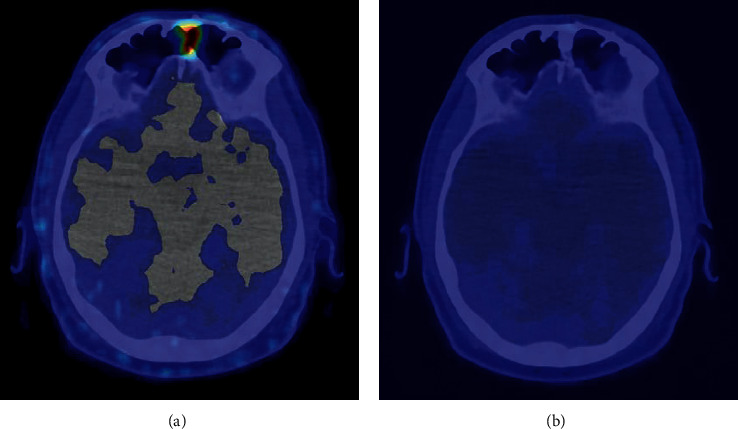
^68^Ga-DOTATATE PET-CT images showing complete response of the recurrent ossifying fibromyxoid tumor in the left frontal sinus after surgery and adjuvant radiotherapy. ^68^Ga-DOTATATE PET-CT images before treatment (a) and after treatment (b).

Figure 2 shows the complete disappearance of the recurrent ossifying fibromyxoid tumor in the left frontal sinus after surgery and adjuvant radiotherapy.

After 60 months from treatment, the patient is free from both tumor recurrence and TIO.

## 3. Discussion

The role of radiotherapy in the multidisciplinary treatment of TIO is far to be defined due to the rarity of this condition. This report described the case of a patient with TIO caused by a multiple recurrent ossifying fibromyxoid tumor, who was cured by surgery and postoperative radiotherapy. To our knowledge, only eight cases of radiotherapy treatment for TIO have been reported so far [3, 4, 6, 7, 9].  Table 2 reports characteristics of all published cases. Five patients were male, and three were female. Patients' age ranged between 43 and 67 years. The cause of oncogenic osteomalacia was a phosphaturic mesenchymal tumor in most cases, and most tumors were in the head region. All patients but one had tumor recurrence after surgery, that was multiple in four cases. In patients with tumor recurrence after previous surgery, radiotherapy was used alone (two patients) or after either partial (four cases) or complete (present case) tumor reresection. A patient who refused surgery for an intracranial lesion underwent radiotherapy as the sole treatment modality.

External beam radiotherapy was used in six cases. Peptide receptor radiotherapy (PPRT) was used in the other two cases. External beam radiation treatment was poorly detailed in published reports. When specified, the total radiation dose ranged between 45 and 66 Gy, and treatment was delivered with conventional fractionation. All patients who underwent radiotherapy had complete remission of TIO. Particularly, three patients who had more than three years of follow-up experienced long-term tumor control and TIO remission.

In conclusion, external beam radiotherapy might provide long-term tumor control and TIO remission, thus being a possible option whenever surgery is unfeasible or unsuccessful.

## Figures and Tables

**Table 1:** Dose received by organs at risk.

Organ at risk	Maximum dose (Gy)	Mean dose (Gy)
Left eye	41.7	5.6
Right eye	26.8	6.0
Left lens	5.9	3.3
Right lens	5.5	3.6
Optic chiasm	5.8	3.1
Left optic nerve	14.7	6.2
Right optic nerve	16.8	6.9
Brain	62.0	7.4

**Table 2:** Details of all published cases of radiotherapy use for TIO.

Reference	Case	Age	Sex	Histopathology	Lesion site	Previous surgery	Radiotherapy indication	Radiotherapy treatment modality	EBRT, total dose (Gy)	EBRT, fraction dose (Gy)	EBRT technique	Follow-up time (months)	Local tumor control	Status osteomalacia
Uramoto, 2009	1	48	M	Malignant PMTMCT	Tongue	Yes, 1 time	Unresectable recurrence of the lesion	EBRT	66	Not specified	Not specified	24	Unknown	Cured
Tarasova, 2013	2	67	F	Unknown	Intracranial	No	Surgery refusal	EBRT	60	Not specified	SRT	12	Unknown	Cured
Hautmann, 2014	3	45	M	PMTMCT	Distal tibia	Yes, 1 time	Postoperative, partial resection	EBRT	45	1.8	Not specified	34	Complete remission	Cured
Basu, 2016	4	53	F	Benign giant-cell tumor	Base of the skull	Yes, multiple	Unresectable recurrence of the lesion	PPRT (peptide receptor radiotherapy)	-	-	-	3	Partial response	Cured
Shah, 2019	5	46	M	Hemangiopericytoma	Left petrous tumor	Yes, 1 time	Postoperative, partial resection	EBRT	Not specified	Not specified	Not specified	96	Complete remission	Cured
	6	53	F	PMTMCT	Base of the skull	Yes, multiple	Postoperative, partial resection	PPRT (peptide receptor radiotherapy)	-	-	-	13	Stable disease	Cured
	7	60	M	PMTMCT	Left ethmoid sinus	Yes, multiple	Postoperative, partial resection	EBRT	54	1.8	IMRT	36	Unknown	Cured
Present case	8	52	M	Ossifying fibromyxoid tumor	Frontal sinus	Yes, multiple	Postoperative, complete resection	EBRT	60	2	VMAT	55	Complete remission	Cured

PMTMCT, phosphaturic mesenchymal tumor mixed connective tissue type; EBRT, external beam radiotherapy; PPRT, peptide receptor radiotherapy; SRT, stereotactic radiotherapy; IMRT, intensity-modulated radiotherapy, VMAT, volumetric modulated arc therapy.

## Data Availability

The data used to support the findings of this study are included within the article.
